# Analysis of prognostic model based on immunotherapy related genes in lung adenocarcinoma

**DOI:** 10.1038/s41598-022-26427-0

**Published:** 2022-12-21

**Authors:** Peng Zhang, Wenmiao Wang, Lei Liu, HouQiang Li, XinYu Sha, Silin Wang, Zhanghao Huang, Youlang Zhou, Jiahai Shi

**Affiliations:** 1 Department of Thoracic Surgery, Nantong Key Laboratory of Translational Medicine in Cardiothoracic Diseases, and Research Institution of Translational Medicine in Cardiothoracic Diseases in Affiliated Hospital of Nantong University, Nantong, 226019 Jiangsu China; 2 Graduate School, Dalian Medical University, Dalian, 116000 Liaoning China; 3 Research Center of Clinical Medicine, Affiliated Hospital of Nantong University, Nantong, 226019 Jiangsu China; 4 School of Public Health, Nantong University, Nantong, 226019 Jiangsu China

**Keywords:** Cancer, Immunology, Oncology

## Abstract

Lung cancer is one of the most common malignant tumors, and ranks high in the list of mortality due to cancers. Lung adenocarcinoma (LUAD) is the most common subtype of lung cancer. Despite progress in the diagnosis and treatment of lung cancer, the prognosis of these patients remains dismal. Therefore, it is crucial to identify the predictors and treatment targets of lung cancer to provide appropriate treatments and improve patient prognosis. In this study, the gene modules related to immunotherapy were screened by weighted gene co-expression network analysis (WGCNA). Using unsupervised clustering, patients in The Cancer Genome Atlas (TCGA) were divided into three clusters based on the gene expression. Next, gene clustering was performed on the prognosis-related differential genes, and a six-gene prognosis model (comprising *PLK1, HMMR, ANLN, SLC2A1, SFTPB,* and *CYP4B1*) was constructed using least absolute shrinkage and selection operator (LASSO) analysis. Patients with LUAD were divided into two groups: high-risk and low-risk. Significant differences were found in the survival, immune cell infiltration, Tumor mutational burden (TMB), immune checkpoints, and immune microenvironment between the high- and low-risk groups. Finally, the accuracy of the prognostic model was verified in the Gene Expression Omnibus (GEO) dataset in patients with LUAD (GSE30219, GSE31210, GSE50081, GSE72094).

## Introduction

Lung cancer is one of the most common malignant tumors, and the main cause of cancer-related death worldwide^1^. Among these cancers, LUAD is the most common histological subtype, accounting for more than 40% of the incidence rate of lung cancer^2^. Most patients with LUAD have advanced or extensive metastasis at the time of diagnosis, and the prognosis is very poor^3^. Despite advances in medical technology and improved clinical outcomes with surgery, radiotherapy, and chemotherapy, the prognosis of these patients remains unsatisfactory. The development of immune checkpoint inhibitors has made immunotherapy for LUAD effective, and improved the survival rate of patients with advanced LUAD. Nevertheless, only few patients can benefit from immunotherapy, and the toxic and adverse effects of immunotherapy continue to remain a challenge^4,5^. As a result, it is imperative to study the tumor microenvironment (TME) and possibilities of immunotherapy for the precise treatment of patients with LUAD.

Histopathologically, LUAD is characterized by the infiltration of a large number of different kinds of immune cells, including B cells, T lymphocytes, natural killer (NK) cells, macrophages, dendritic cells (DC), and Myeloid-derived suppressor cells (MDSC)^6^. These immune cells play different functions and create a microenvironment for the development of lung cancer. Studies have shown that immune microenvironment plays an important role in the incidence and development of tumors^7^. Immune cells, mesenchymal cells, and the extracellular matrix constitute the main components of the TME and are decisive in determining tumor invasiveness^8^. In addition, some studies have pointed out that some key chemokine networks in TME can recruit different immune cells into TME, enhance different mechanisms, and thus promote or inhibit tumor progression. They have also clarified the relationship between TME and the occurrence and development of immune cells and tumors, thus laying a solid foundation for the immunotherapy of malignant tumors and provided broad-ranging therapeutic targets^9^.

Immunotherapy provides a new strategy for patients with advanced adenocarcinoma. immune checkpoint receptor blockers, such as anti-programmed cell death protein 1 (PD-1) and anti-cytotoxic T lymphocyte associated protein 4 (CTLA-4), enhance anti-tumor immune response by targeting T lymphocyte regulatory pathways, and have achieved great progress^10^.

In this study, the gene co-expression network, WGCNA was constructed to screen gene modules related to immunotherapy. A total of 19 modules were identified, and the module with the strongest correlation was magenta. Prognosis-related genes were screened by difference analysis and univariate Cox regression. The patients were divided into three clusters (cluster A, cluster B, and cluster C) through consensus classification. The survival of cluster B was greater than that of clusters C and A. Subsequently, 125 differentially expressed genes (DEGs) were identified among the three clusters. Through univariate Cox regression, 78 DEGs related to the prognosis were screened. LASSO analysis identified six key genes that were then used to build a prognosis model. Survival analysis indicated that patients with high-risk scores had poorer prognosis. Follow-up studies also showed significant differences in the tumor immune microenvironment, tumor mutation load, immunotherapy, and immune checkpoints, between the high-risk and low-risk score groups. Finally, the efficacy of this prognostic model was successfully verified in the data set of four external cohorts (GSE30219, GSE31210, GSE50081, GSE72094).

## Materials and methods

The study is in accordance with relevant guidelines and regulations.

### Data download

The transcriptome data based on RNA SEQ of lung LUAD patients and the corresponding clinical data of LUAD patients were downloaded from TCGA database, including the FPKM value of gene expression in 539 LUAD samples and 59 normal samples (fpkm; transcripts per kilobase of mapping readings per million), followed by the conversion of FPKM values into TPM values for data processing. Download the data of four queues of patients with LUAD from GEO database, GSE30219 (n = 85), GSE31210 (n = 226), GSE50081 (n = 127) and GSE72094 (n = 398).

### Construction of weighted gene coexpression network and identification of modules related to immunotherapy in LUAD patients

Weighted gene coexpression network analysis is a system biology method, which can be used to find highly correlated gene clusters (modules)^11^. In this study, WGCNA was used to identify the modules related to immunotherapy. Select soft threshold β = 5 (scale-free r2 = 0.9) to construct a co expression network. Then we transform adjacency matrix into topological overlap matrix to quantitatively describe similarity. Next, we used the cutreedynamic function to execute the gene hierarchical clustering tree and finally identified 19 coexpression modules.

### Extraction of differential genes and prognosis related genes

"limma" package was used to identify apoptosis related genes differentially expressed between LUAD and normal tissues in TCGA database. The screening criteria are error detection rate (FDR) < 0.05, |logfc|> 0.5. Then, univariate Cox regression analysis was used to screen the prognoses related DEG.

### Consensus clustering

The prognostically related DEGs are clustered. The number and stability of the clusters are determined by the consensus clustering algorithm using the "ConsensusClusterPlus" package, which is repeated 1000 times to ensure the classification stability. The prompt function is used for principal component analysis. Heat maps and Kaplan Meyer (km) curves are drawn using R packages "Heatmap", "Survivminer" and "Survival".

### Model construction and validation

The consensus clustering algorithm divides the patients into three subtypes. Next, we use the R package "limma" to identify the differentially expressed genes among the subtypes (|logfc|> 1). After using univariate Cox regression analysis to screen DEGs related to prognosis, Lasso Cox analysis was used to construct a prognostic model with 6 genes characteristics. Use the "survminer" package to determine the median cutoff. Kaplan Meier survival curve was used to determine the overall survival time (OS) of patients with different subtypes. Time dependent ROC curve was used to evaluate the validity and accuracy of the model. Finally, the accuracy of the prognostic model is verified in the GEO datasets.

### Calculate the immune score of TME

The immune score, stromal score, estimated score and tumor purity were obtained according to the transcriptomic spectrums expression, and the tumor purity was calculated by "estimate" R package.

### Enrichment analysis

For differential genes in high-risk and low-risk groups,Gene ontology(GO), Kyoto Encyclopedia of Genes and Genomes (KEGG) pathway, Gene Set Enrichment Analysis (GSEA) and Gene Set Variation Analysis (GSVA) were used to evaluate biological effects. In order to further study the potential regulatory mechanism of tumor immune cell infiltration, a single sample gene set enrichment analysis (ssGSEA) was performed to evaluate the infiltration abundance between high-risk and low-risk groups.

### Statistical analysis

All statistical analyses were performed by the R statistical language (version 4.0.3). Wilcoxon test and Kruskal Wallis test were used to compare two groups and more than two groups respectively. Kaplan Meier plotter was used to plot the prognosis survival curve, and log rank test was used to evaluate the significance of statistical difference. Spearman test is used for correlation analysis and calculation of correlation coefficient. All statistical tests were bidirectional, and P values less than 0.05 were considered statistically significant (* P < 0.05, * P < 0.01, * P < 0.001).

## Results

### WGCNA and modules significance calculation

**Figure 1:**
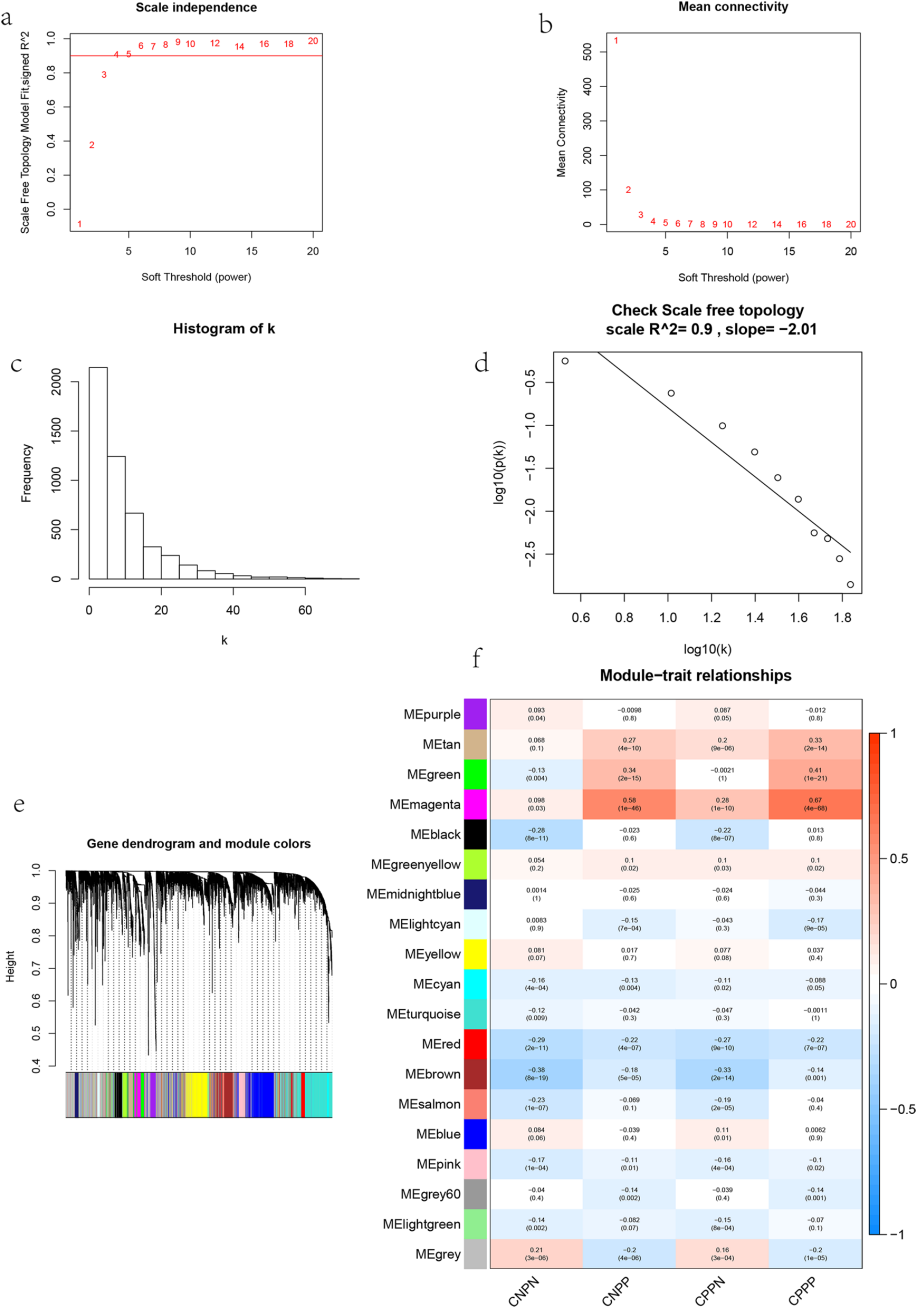
Sample dendrogram and soft-thresholding values estimation. (**a**) Scale free index analysis of coexpression module genes under different soft thresholds. (**b**) Average connectivity analysis of coexpression module genes under different soft thresholds. (**c**, **d**) β = Scale free topology at 5. (**e**) Gene clustering tree based on topological overlap. (**f**) heat map of correlation between 19 module genes and different characteristics. CNPN, CTLA4_Negative_PD1_Negative; CNPP, CTLA4_Negative_PD1_Positive; CPPN, CTLA4_Positive_PD1_Negative; CPPP, CTLA4_Positive_PD1_Postive.

In order to ensure high scale independence, we use soft threshold β Set to 5 (scale-free R2 = 0.9, Fig. 1a, b) to obtain β The adjacency matrix and topological overlap matrix (Fig. 1c, d) were constructed, the gene expression matrix of 5000 pretreatment genes was analyzed by WGCNA (Table S1), and the correlation coefficient between each module and the samples related to the characteristics of CNPN, CNPP, CPPN and CPPP was calculated. A total of 19 modules were obtained (Fig. 1e, f). From the module feature correlation heat map, we found that magenta module has the highest correlation with CNPN, CNPP, CPPN and CPPP (CNPN: cor = 0.098; P = 0.03. CNPP: cor = 0.58; P = 1e−46. CPPN: cor = 0.28; P = 1e−10. CPPP: cor = 0.67; P = 4e−68).

### Extraction of differential genes and prognosis related genes

**Figure 2:**
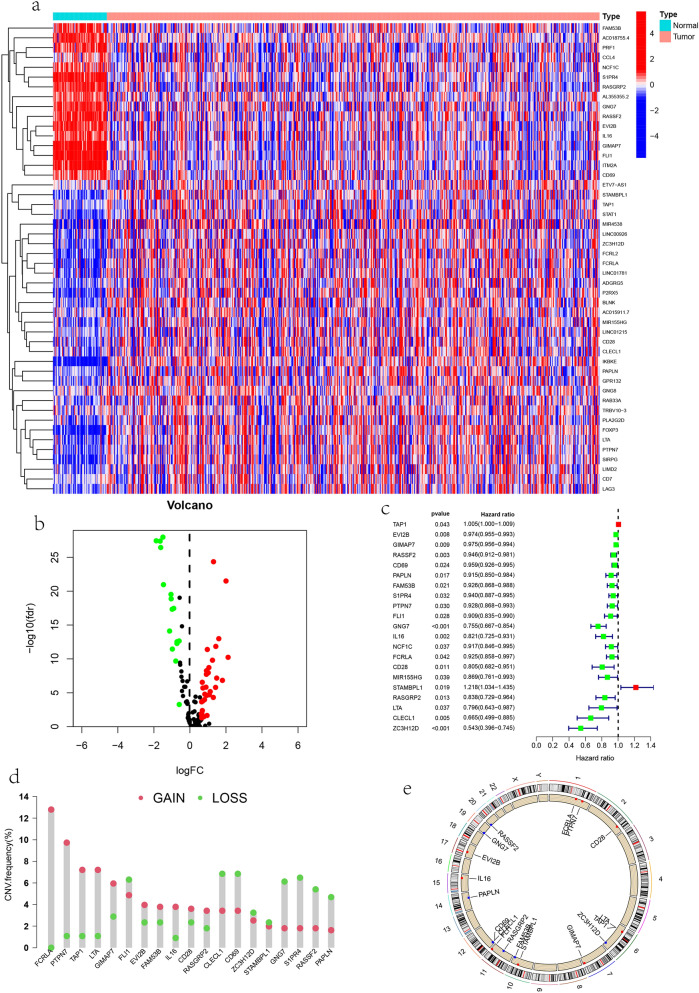
Extraction and CNV of differential genes. (**a**) Heat map of differential gene expression in normal and tumor tissues, heat map of prognosis related genes between normal (N, bright blue) and tumor tissues (T, red) (blue: low expression level; red: high expression level); (**b**) volcano map shows the regulation of differential genes in lung adenocarcinoma(LUAD) and normal tissues in The Cancer Genome Atlas (TCGA) cohort (green: down regulation; red: up regulation); (**c**) the forest map of genes related to prognosis was screened by univariate Cox analysis; (**d**) copy number variation (CNV) frequencies of prognosis related genes in the TCGA cohort. The height of the column represents the change frequency. The green dot represents the missing frequency. The red dot represents the amplification frequency. (**e**) Location of CNV changes in genes on 23 chromosomes.

By comparing the differential expression of magenta module genes in normal tissues and LUAD tissues, 48 differential expression genes were identified. The heat map shows the expression of each differential gene in each sample (Fig. 2a). The volcano map shows the up regulation and down regulation of differential genes (Fig. 2b). Univariate Cox regression analysis was used to screen 21 prognostically related DEGs (Table S2), as shown in the forest diagram (Fig. 2c). Gene mutation (Fig. S1) shows that among 561 samples, 75 had mutations in central regulatory factors, with a frequency of 13.37%. It was found that IL16 had the highest mutation frequency, followed by *FCRLA, FLI1, RASSF2, GIMAP7, EVI2B, PAPLIN, S19R4, RASGRP2.* The rest of the regulatory factors did not show any mutations in the sample. The investigation of Copy number variation(CNV) frequency(Fig. 2d) showed that 19 central regulatory factors had copy number variation, *FCRLA, PTPN7, TAP1, LTA, GIMAP7, EVI2B, FAM53B, IL16, CD28* focused on the amplification of copy number, and FCRLA had the highest amplification frequency. *RASSF2, CD69, PAPPIN, S1PR4, FLI1, GNG7, STAMBPL1, CLECL1, ZC3H12D* focused on the deletion of copy number. The deletion frequencies of CLECL1 and CD69 were the highest. In addition, the altered position of the central regulator CNV on the chromosome is also shown (Fig. 2e).

### Consensus clustering based on prognostic related genes

**Figure 3:**
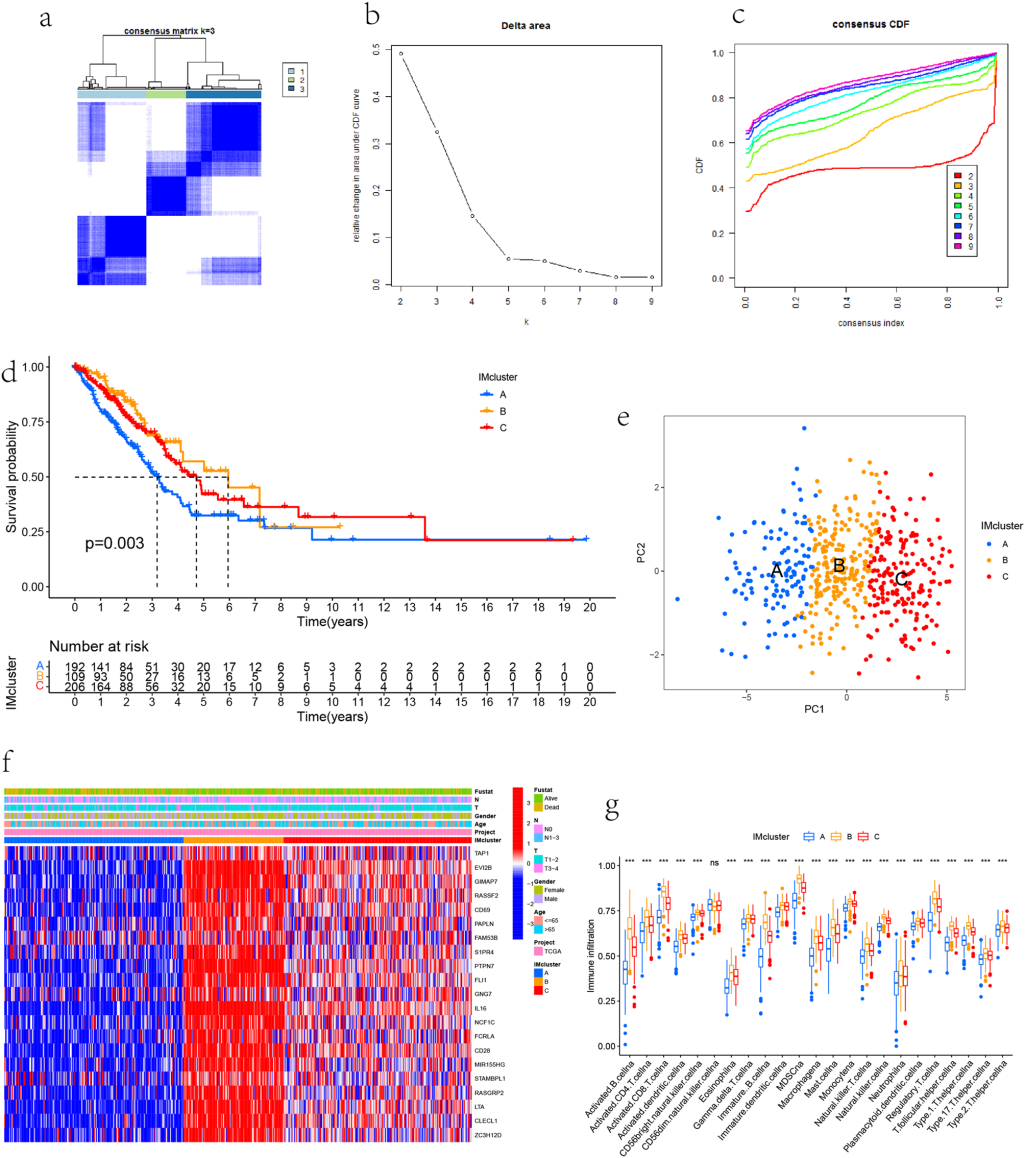
OS curve, expression and immune cell infiltration among clusters. (**a**–**c**) Consensus clustering heat map of lung adenocarcinoma (LUAD) patients when k = 3. (**b**) Delta area curve of consensus clustering represents the relative change of area under the cumulative distribution function (CDF) curve. (**c**) For consensus clustering CDF with k = 2–9. (**d**) Kaplan Meier OS curve among the three clusters. (**e**) Principal component analysis (PCA) showed the sample distribution of the three clusters. (**f**) The expression of differentially expressed genes in the three clusters and their clinicopathological characteristics. Red and blue represent high and low expressions of genes respectively. (**g**) The degree of infiltration of immune cells among the three clusters. The P value is displayed as: ns: not significant *P < 0.05; **P < 0.01; ***P < 0.001.

Unsupervised clustering of LUAD patients with different expression patterns of 21 immune prognosis related genes was carried out using the R package of consensusclusterplus. In order to ensure the stability of classification, 1000 iterations were carried out, and the resampling rate was 80%. The cumulative distribution function (CDF) curve is used to determine the number of clusters and determine that k = 3 has the best cluster stability from k = 2 to 9 according to the s imilarity (Fig. 3a–c). Finally, three different clusters (A, B, C) were identified, and the OS curve indicated the significant survival advantage of cluster B in the three main clusters (P = 0.003, Fig. 3d). Then Principal component analysis (PCA) was used to determine the sample distribution of the three clusters (Fig. 3e). The Heatmap showed high expression of prognosis related genes in cluster B and low expression in cluster A (Fig. 3f). ssGSEA analysis showed that there were significant differences in the degree of immune cell infiltration among the three clusters (Fig. 3g). Except for the unintentional expression of cd56dim.natural.killer.cellna, the expression of the other 22 immune cells was the lowest in cluster A and the highest in cluster B, such as activated B. cellna (P < 0.001), Activated. CD4. T. cellna (P < 0.001), Activated. CD8. T. cellna (P < 0.001), Eosinophilna (P < 0.001), MDSCna (P < 0.001), Macrophagena (P < 0.001), Mast. cellna (P < 0.001), Monocytena (P < 0.001), Natural. killer. Cellna (P < 0.001), neutrophilna (P < 0.001), among others. The immune cell infiltration level of cluster A was the lowest, indicating that the immune response of cluster A was the lowest, which is consistent with the poor survival results. The immune cell infiltration level of cluster B was the highest, indicating that the immune response of cluster B was the highest, which is consistent with the better survival results. To explore the differences in biological behavior among different clusters, we performed KEGG gene set variation analysis (GSVA) (Fig. S2a–2d). The results showed that the OXIDATIVE_PHOSPHORYLATION and PARKINSONS_DISEASE were mainly enriched in cluster A compared with cluster B. B_KILLER_CELL_MEDIATED_CYTOTOXICITY, T_CELL_RECEPTOR_SIGNALING_PATHWAY, B_CELL_RECEPTOR_SIGNALING_PATHWAY were mainly enriched in cluster B. Cluster A compared to cluster B, PRIMARY_IMMUNODEFICIENCY, INTESTINAL_IMMUNE_NETWORK_FOR_IGA_PRODUCTION, HEMATOPOIETIC_CELL_LINEAGE, ALLOGRAFT_REJECTION, AUTOIMMUNE_THYROID_DISEASE were mainly highly expressed in cluster B and low expressed in cluster A. cluster B compared to cluster C, PRIMARY_IMMUNODEFICIENCY, INTESTINAL_IMMUNE_NETWORK_FOR_IGA_PRODUCTION, AUTOIMMUNE_THYROID_DISEASE, ALLOGRAFT_REJECTION, JAK_STAT_SIGNALING_PATHWAY, CYTOKINE_CYTOKINE_RECEPTOR_INTERAVTION were mainly highly expressed in cluster B, and cluster C was mainly related to ARGININE_AND_PROLINE_METABOLISM, GLYCOSYLPHOSPHATIDYLINOSITOL_GPI_ANCHOR_BIOSYNTHESIS ALZHEIMERS_DISEASE, HUNTINGTONS_DISEASE, PARKINSONS_DISEASE.

### Consensus clustering based on DEG among different clusters

**Figure 4:**
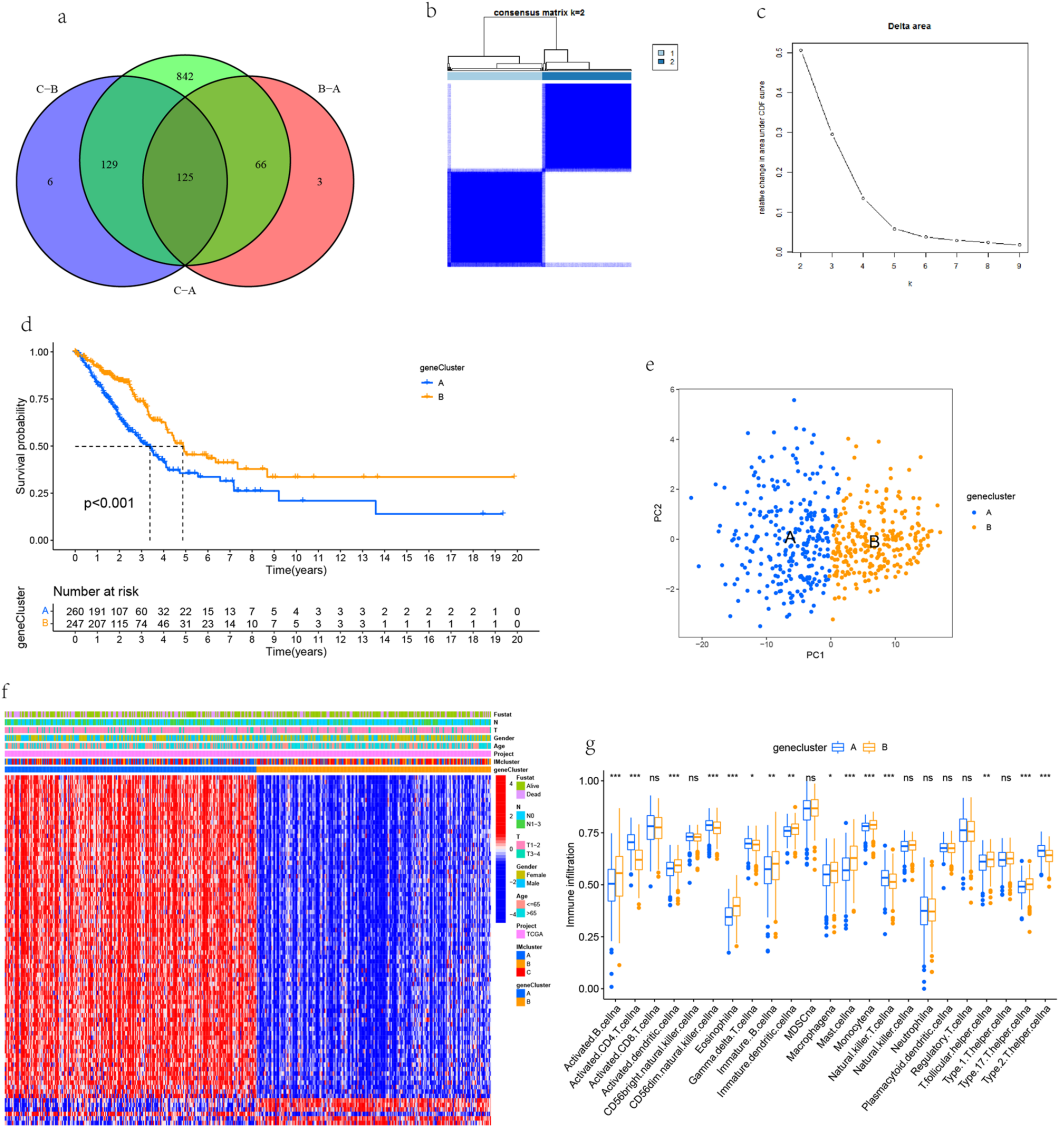
OS curve, clinical correlation and immune cell infiltration among geneclusters. (**a**) Venn diagram between the three clusters. (**b**, **c**) The genes were divided into two clusters according to the consensus clustering matrix (k = 2). (**d**) Kaplan Meier OS curve for two clusters. (**e**) Principal component analysis (PCA) shows the sample distribution of the two clusters. (**f**) The Heatmap showed the clinicopathological features of genes with different prognosis. (**g**) The degree of infiltration of immune cells between the two clusters. The pvalue was displayed as: ns: not significant * P < 0.05; ** P < 0.01; *** P < 0.001.

Based on 125 DEGs (Fig. 4a, Table S3) of the intersection of three clusters, 78 prognosis related genes (Table S4) were screened out through univariate analysis for unsupervised cluster analysis. In order to ensure the stability of classification, 1000 iterations are carried out, and the resampling rate is 80%. The cumulative distribution function (CDF) curve is used to determine the number of clusters and determine that k = 2 has the best cluster stability from k = 2 to 9 according to the s imilarity (Fig. 4b, c). Finally, two different clusters (A, B) were identified. Kaplan Meier OS curves for both clusters showed that patients with gene cluster B had better prognosis (P < 0.001) (Fig. 4d). Then the PCA algorithm is used to confirm that the samples of the two risk groups are distributed separately (Fig. 4e). The Heatmap shows the clinicopathological features of prognostically relevant DEGs (Fig. 4f). ssGSEA analysis showed that there were significant differences in the degree of immune cell infiltration between the two clusters (Fig. 4g). Activated. CD4. T. cellna (P < 0.001) , CD56d im. natural. killer. cellna (P < 0.001), Natural. killer. T. cellna (P < 0.001), Type. 2. T. helper. cellna (P < 0.001), Gamma. delta. T. Cellna (P < 0.05) are mainly enriched in cluster A. Activated B. cellna (P < 0.001), Activated. dendritic. cellna (P < 0.001), Eosinophilna (P < 0.001), Mast. cellna (P < 0.001), Monocytena (P < 0.001), Type. 17. T. helper. cellna (P < 0.001), immature. B. cellna (P < 0.01), immature. dendritic. cellna (P < 0.01), T. follicular. helper. Cellna (P < 0.01) and macrophagena (P < 0.05) are mainly enriched in cluster B.

### Construction of prognosis model

**Figure 5:**
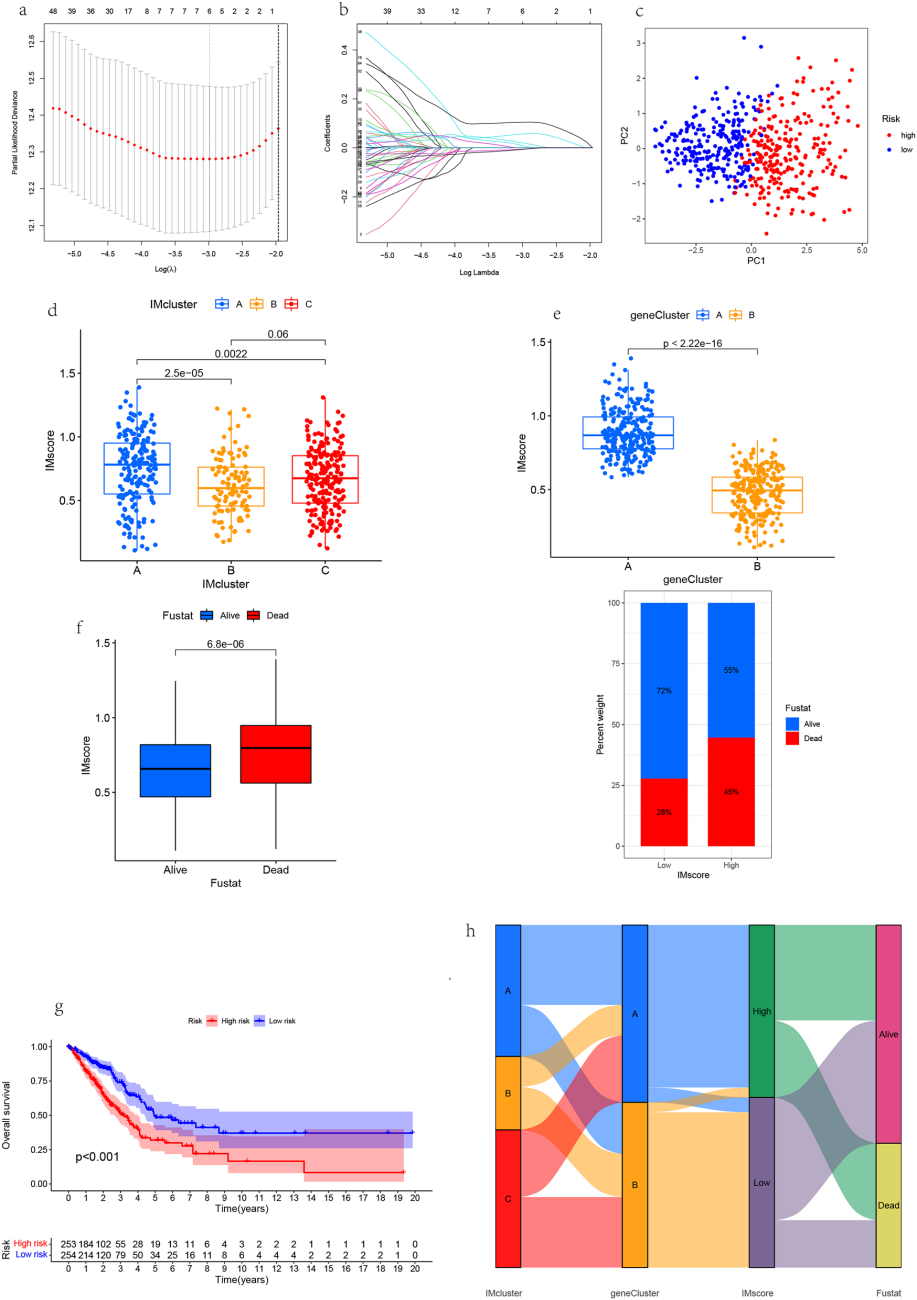
Lasso regression analysis was used to construct prognostic gene features. (**a**) Least absolute shrinkage and selection operator (Lasso) coefficient spectrum of 6 potential prognostic genes related to immunity. (**b**) The best parameters in lasso regression were selected by 10 × cross validation. Lasso, min imum absolute contraction and selection operator Cox regression model. (**c**) Principal component analysis (PCA) showed the sample distribution of different risk score groups. (**d**) IMscore among different cluster. (**e**) IMscore among different genecluster. (**f**) The relationship between IMscore and survival status. (**g**) Kaplan Meier OS curve between high risk group and low risk group (P < 0.001). (**h**) Sankey diagram showing the relationship between IMcluster, genecluster, IMscore, and survival status.

In order to avoid over fitting, Lasso Cox regression analysis was performed on 78 differential genes related to prognosis, and Lasso coefficient spectra of 6 potential prognostic genes related to immunity were established (Fig. 5a). Then the optimal penalty parameters of lasso model were determined through ten-fold cross validation (λ) (Fig. 5b), find the key genes with the strongest correlation through dimension reduction, and calculate the relative coefficient of genes (Table S5). Finally, six genes, *Plk1,HMMR, ANLN,SLC2A1, SFTPB,CYP4B1* were established to construct the prognosis model and score. We named it "IMscore". Risk scoring formula = (*Plk1*mRNA level *0.05682) + (*HMMR*mRNA level *0.00878) + (*ANLN*mRNA level *0.10474) + *SLC2A1*mRNA level *0.01988) + (*SFTPB*mRNA level *− 0.00501) + *CYP4B1*mRNA level *−0.00608). Among them, 2 genes are protective factors (*SFTPB,CYP4B1*), and 4 genes are risk factors (*Plk1,HMMR, ANLN,SLC2A1*). Calculate the risk score for each patient according to the formula. According to the optimal threshold, patients were divided into high-risk and low-risk groups (Table S6). PCA showed (Fig. 5c) that patients with different risks could be divided into two groups. There were differences in IMscore among different subtypes. IMcluster A has the highest risk value and IMcluster B has the lowest risk value. The prognosis of high scores is poor, which is consistent with the previous studies (Fig. 5d). In genecluster, there were also differences in IMscore. The risk value of genecluster A was greater than that of cluster B, and the prognosis of cluster A is worse, which is consistent with the previous studies (Fig. 5e). Combining the IMscore with the clinical survival status, it was found that the IMscore of the dead patients was much larger than that of the living patients, and the patient mortality increased with the increase of the risk value (Fig. 5f). Survival analysis showed that there were significant differences between the high-risk group and low-risk group, and the survival of the high-risk group was worse (P < 0.001, Fig. 5g). Finally, the IMscore, genecluster, high-risk, low-risk and survival status were connected through the Sankey diagram. Most of the clusterB with the best prognosis in IMscore belong to geneclusterB with better prognosis in genotyping, and most of them belong to the low-risk group with better prognosis (Fig. 5h).

### Evaluation of correlation between risk score and clinical characteristics

**Figure 6:**
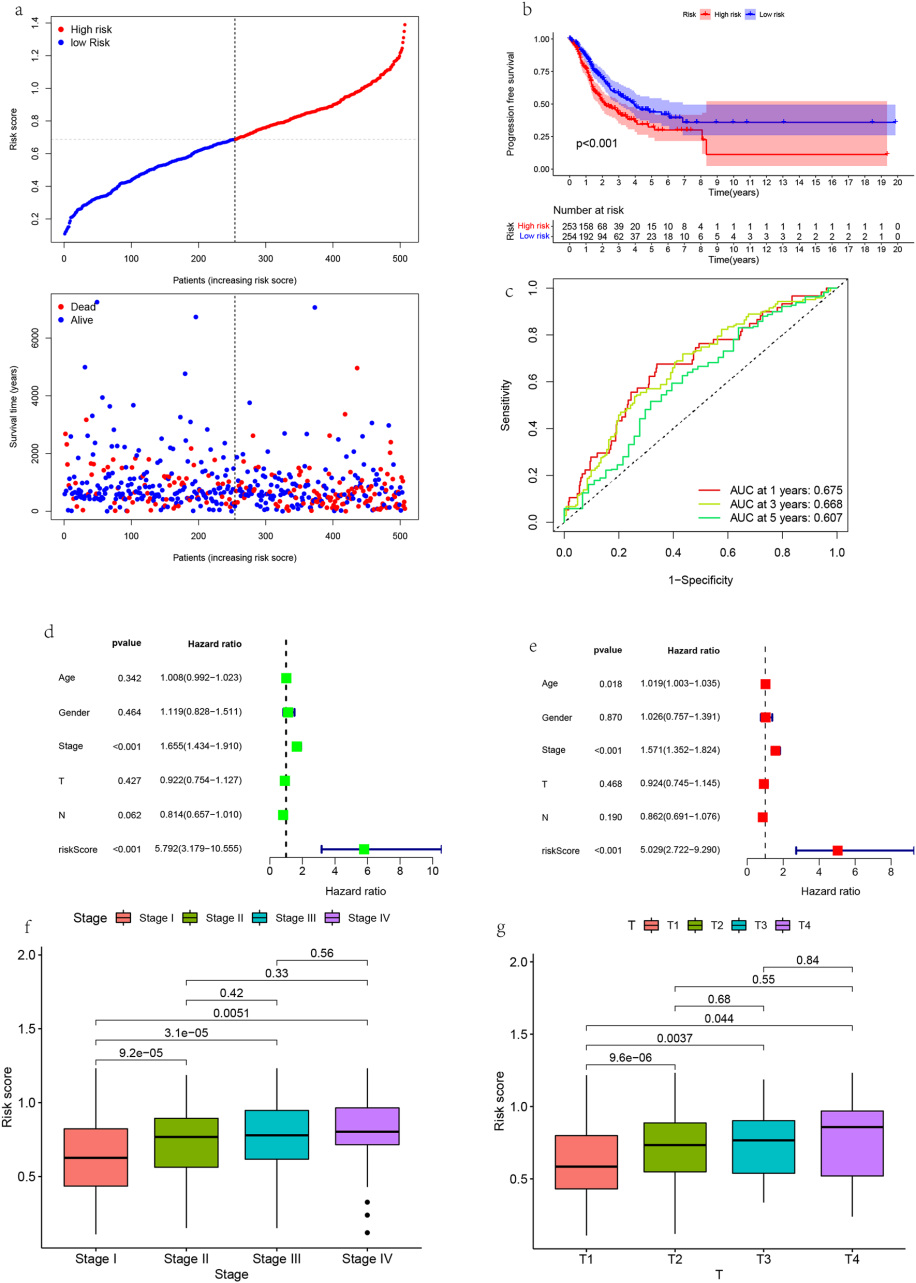
Clinical correlation analysis of prognosis model. (**a**) Risk score distribution and survival status of each patient. (**b**) Progression free survival. (**c**) Receiver operating characteristic (ROC) curve shows the prediction efficiency of risk score. (**d**) Univariate Cox regression analysis in TCGA cohort. (**e**) Multivariate Cox regression analysis in The Cancer Genome Atlas (TCGA) cohort. (**f**), (**g**) Relationship between clinical stages and risk scores.

The risk curve (Fig. 6a) shows that LUAD patients are divided into high-risk and low-risk groups according to the median value of the risk score. The IMscore of the high-risk group is higher than that of the low-risk group. With the increase of the risk value, the number of dead patients increases. The progression free survival showed that the high-risk group was lower than the low-risk group (P < 0.001, Fig. 6b). The predictive effect of OS prognostic characteristics in LUAD patients was evaluated by time-dependent receiver operating characteristic (ROC) curve. The areas under the curve were (AUC) 0.675 in 1 year, 0.668 in 3 years and 0.607 in 5 years (Fig. 6c), indicating that the model has high sensitivity and specificity in predicting the prognosis of LUAD patients. Subsequently, we performed univariate and multivariable Cox analysis based on the risk scores obtained from immune related prognostic characteristics and the main clinical characteristics of LUAD patients in TCGA database. Univariate Cox analysis confirmed that higher stage and risk score were risk factors for HRS > 1 in LUAD patients, P < 0.001 (Fig. 6d). After removing other factors, a further multivariable Cox analysis (Fig. 6e) showed that higher stage and risk score were proved to be independent prognostic factors for OS in LUAD patients (stage HR = 1.571, 95% CI: 1.352–1.824, P < 0.001; risk score HR = 5.029, 95% CI: 2.722–9.290, P < 0.001). Stage stage shows that the risk score increases with the increase of stage, and the risk value of stage IV is the highest (Fig. 6f); T stage indicates that the risk score increases with the increase of stage, and the risk value in T4 stage is the highest (Fig. 6g). Clinical staging showed that the prognostic risk characteristics were closely related to the degree of malignancy.

### Nomograph modeling using clinical characteristics and risk scores

**Figure 7:**
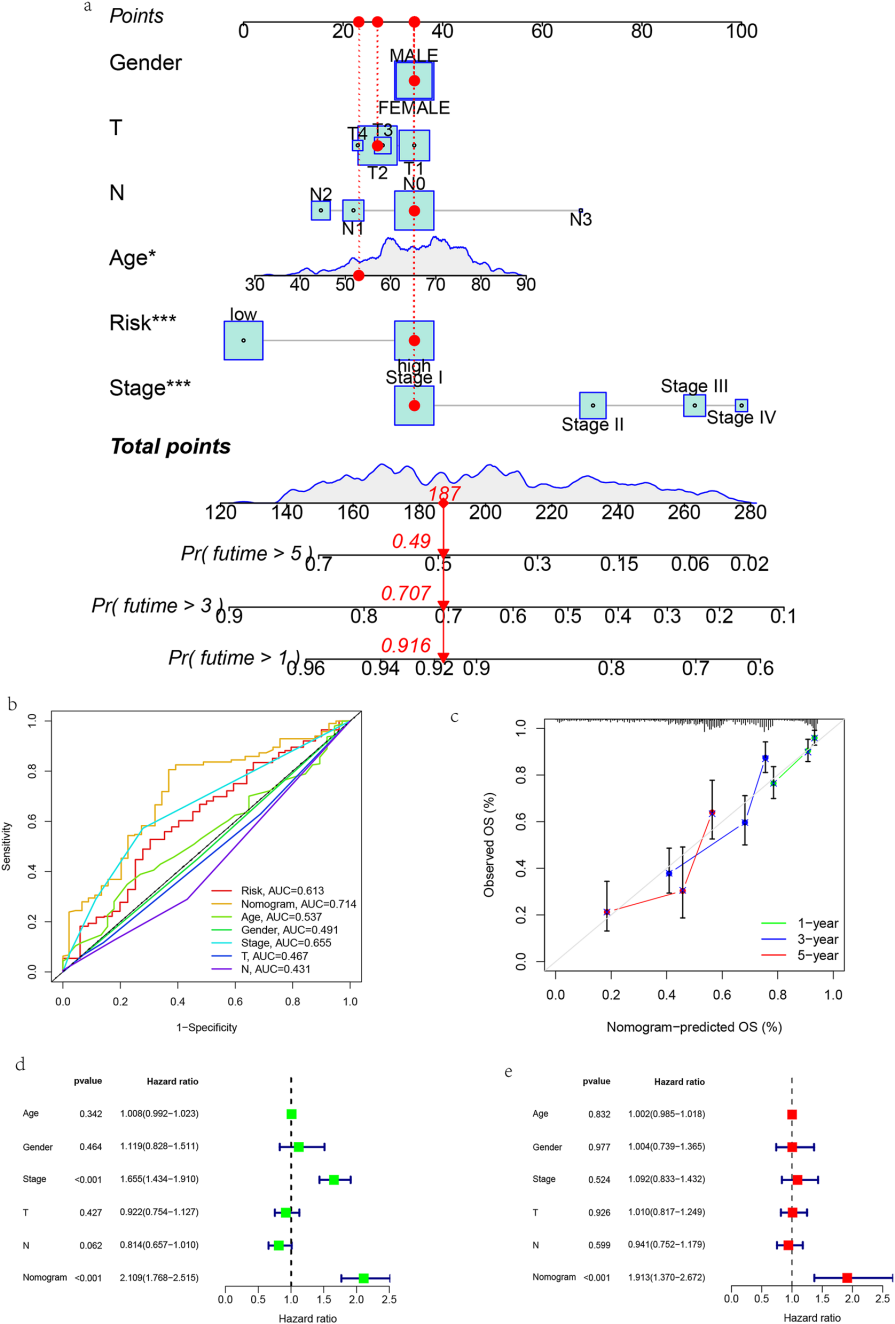
Construction of nomograms. (**a**) Nomograms used to predict the 1, 3, and 5-year overall survival of lung adenocarcinoma (LUAD) patients in the The Cancer Genome Atlas (TCGA) cohort. (**b**) Calibration chart for predicting recurrence in 1, 3 and 5 years. (**c**) Receiver operating characteristic (ROC) curve evaluates the sensitivity of nomograph model. (**d**) Univariate Cox regression analysis in TCGA cohort. (**e**) Multivariate Cox regression analysis in TCGA cohort.

In order to make better use of the prognosis model we constructed, nomograms of 1, 3 and 5-year overall survival of LUAD patients in TCGA database were established based on multivariate Cox analysis (Fig. 7a, Table S7). Calibration charts for the 1-, 3-, and 5-year OS are used to visualize the performance of nomograms (Fig. 7b). The sensitivity of the nomogram model was evaluated by ROC curve. The AUC result of the risk scoring model was 0.714 (Fig. 7c), indicating that the nomogram was the best in predicting the survival of LUAD patients compared with other individual prognostic factors. Then, by univariate Cox analysis, the risk score was a risk factor for HRS > 1 in LUAD patients, P < 0.001 (Fig. 7d). Multivariate Cox analysis showed that the risk score proved to be an independent prognostic factor for OS in LUAD patients (risk score HR = 1.913, 95% CI:1.370–2.672, P < 0.001, Fig. 7e).

### Functional analysis between different risk groups

**Figure 8:**
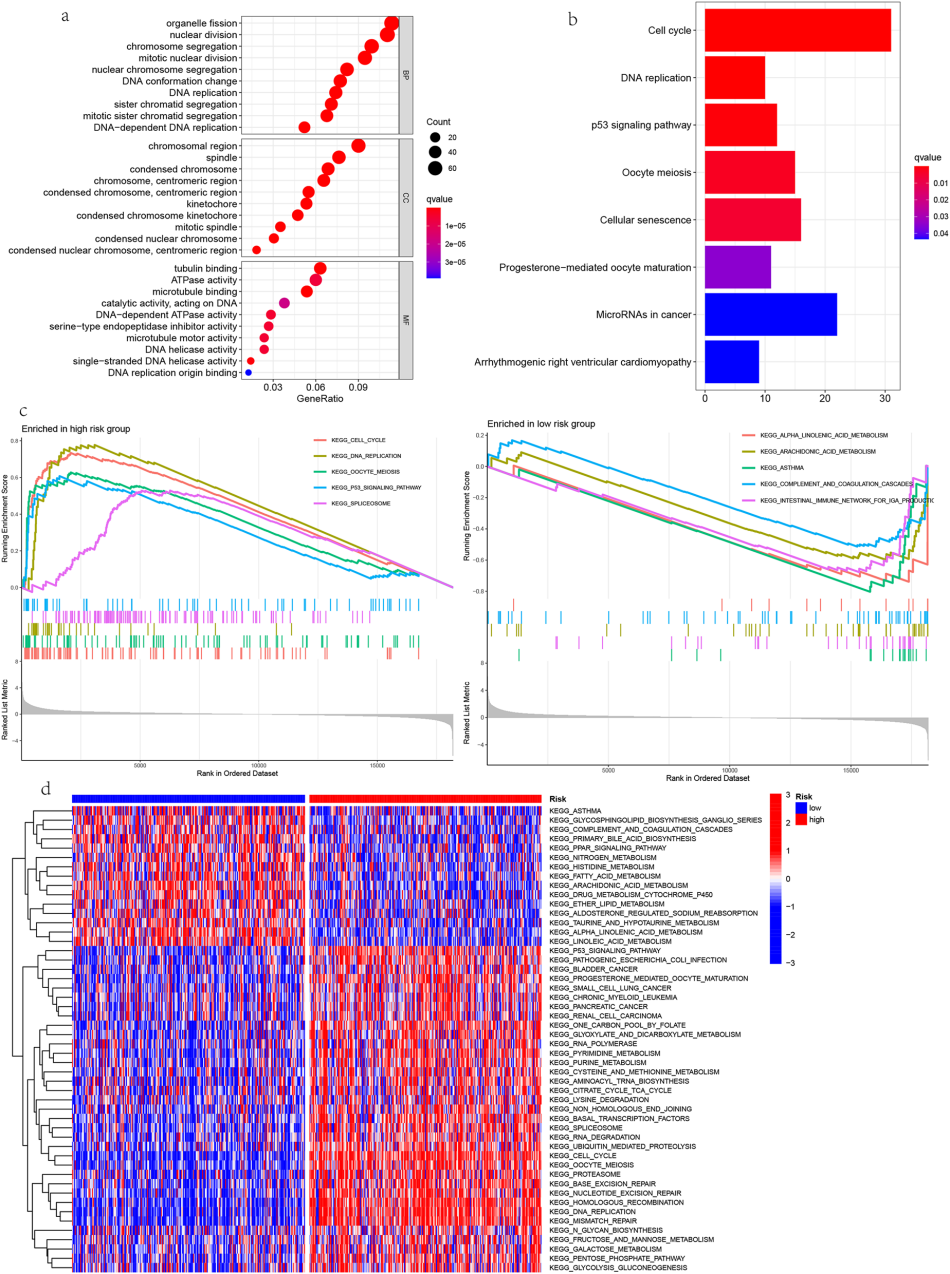
Enrichment analysis. (**a**) The bubble chart shows the Gene ontology (GO) analysis of differential genes between high-risk and low-risk groups based on The Cancer Genome Atlas (TCGA) database. (**b**) The histogram shows Kyoto Encyclopedia of Genes and Genomes (KEGG) analysis of differential genes between high-risk and low-risk groups based on TCGA database. (**c**) Gene Set Enrichment Analysis (GSEA) of differential genes between high-risk and low-risk populations based on TCGA database. The five main up-regulated pathways in the high-risk group (left) and the five main up-regulated pathways in the low-risk group (right). (**d**) Gene Set Variation Analysis (GSVA) of pathway enrichment between high and low risk groups.

In order to study the potential difference of biological function between different risk groups, we conducted GO, KEGG pathway, GSEA and GSVA. GO analysis showed that DEGs between high-risk and low-risk groups were mainly enriched in nuclear division, organelle fission, chromosome segregation. (Fig. 8a, Table S8). KEGG analysis showed that DEGs were mainly enriched in CELL_CYCLE, DNA_REPLICATION and P53_SIGNAL_PATHWAY (Fig. 8b, Table S9). GSEA analysis showed that the high-risk score group was mainly enriched in CELL_CYCLE, DNA_REPLICATION and P53_SIGNAL_PATHWAY, OOCYTE_MEIOSIS, SPLICEOSOME, etc., while the low-risk score group was mainly enriched in ALPHA_LINOLENIC_ACID_METABOLISM, ARACHIDONIC_ACID_METABOLISM, ASTHMA, INTESTINAL_IMMUNE_NETWORK_FOR_IGA_PRODUCTIC,COMPLEMENT_AND_COAGULATION _CASCADE, etc. (Fig. 8c, Table S10). GSVA analysis prompted P53_SIGNALING_PATHWAY, CELL_CYCLE, DNA_REPLICATION, RNA_DEGRADATION, HOMOLOGOUS_RECOMBINATION were mainly enriched in high-risk groups, ASTHMA, PPAR_SIGNALING_PATHWAY, ALPHA_LINOLENIC_ACID_METABOLISM, LINOLEIC_ACID_METABOLISM, COMPLEMENT_AND_COAGULATION_CASCADES and others were mainly enriched in low-risk groups (Fig. 8d, Table S11). Biological function between high and low risk groups in TCGA cohort.

### Correlation analysis between risk score and tumor mutational burden

**Figure 9:**
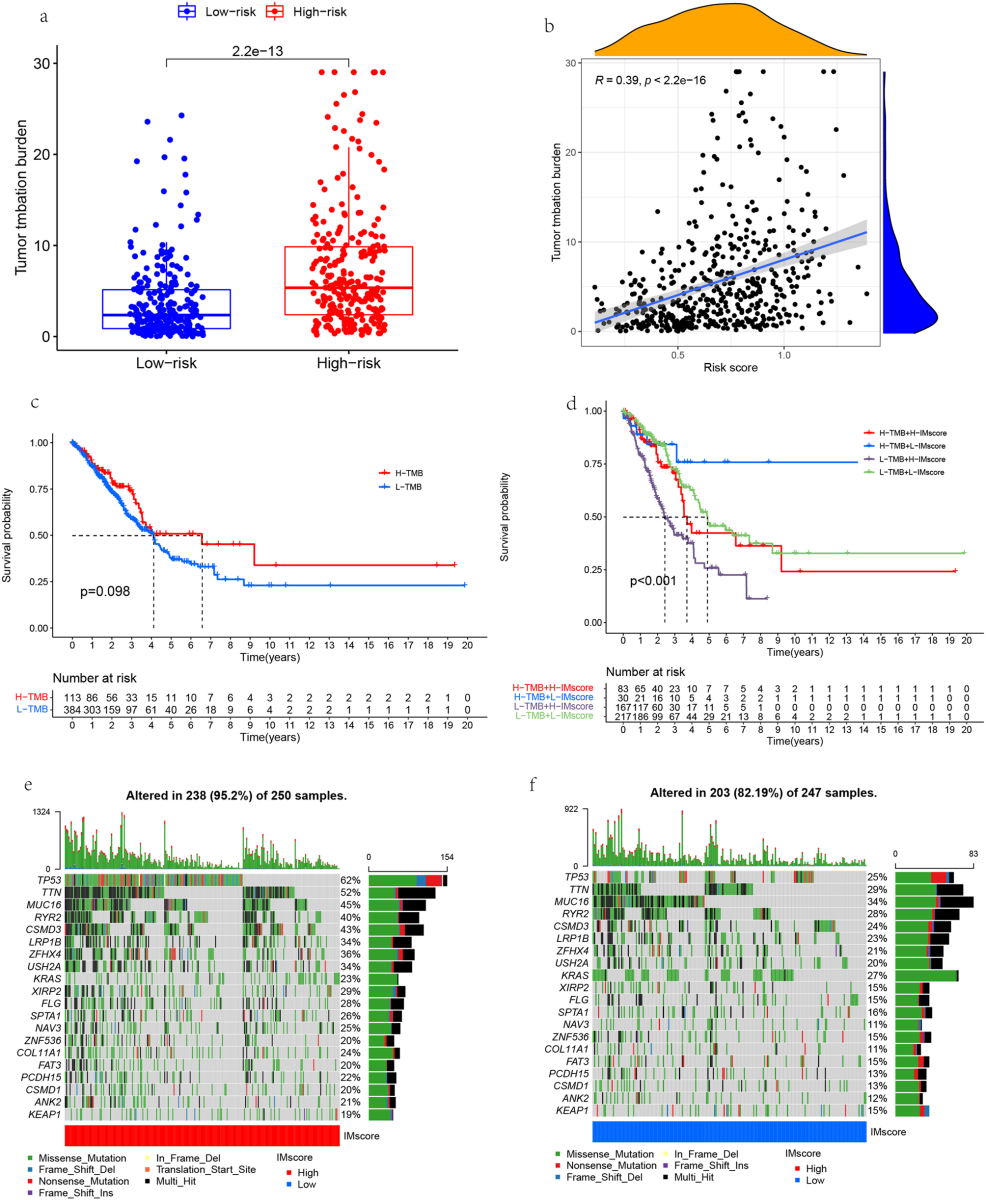
TMB between high and low risk groups. (**a**) Differences in Tumor Mutational Burden (TMB) between high and low risk score groups in the The Cancer Genome Atlas (TCGA) cohort. (**b**) Association between risk score and TMB in TCGA queue. (**c**) Kaplan Meier OS curve of high and low TMB groups. (**d**) Kaplan Meier OS curve of IMscore and TMB. (**e**) High TMB group in TCGA queue. (**f**) Low TMB group in TCGA queue.

There is a significant difference in tumor mutation load between high and low risk scores. The tumor mutation load in the high risk score group is significantly higher than that in the low risk score group (Fig. 9a), and there is a significant positive correlation between tumor mutation load and risk score (Fig. 9b). Survival analysis showed that it was meaningless to study the relationship between high and low tumor mutation load and patient survival alone (Fig. 9c). However, after giving high and low risk scores, OS showed patients with high scores had poor prognosis in both high tumor mutation load group and low tumor mutation load group. Among them, patients with high tumor mutation and low IMscore had the best survival, while patients with low tumor mutation and high IMscore had the worst survival (Fig. 9d). There were differences in gene mutation frequency between high and low IMscore groups. The gene mutation frequency in high IMscore group was higher than that in low IMscore group. The top 20 most significantly mutated genes in the high and low risk score groups were *TP53, TTN, MUC16, RYR2, CSMD3, LRP1B, ZFHX4, USH2A, KRAS, XIRP2, FLG, SPTA1, NAV3, ZNF536, COL11A1, FAT3, PCDH15, CSMD1, ANK2, KEAP1*. In addition, the top five genes with the highest mutation frequency in the high and low risk groups are *TP53, TTN, MUC16, RYR2, CSMD3*. *TP53* mutations are mainly Missense_Mutations and Nonsense_Mutations, while *TTN, MUC16, RYR2, CSMD3* mutations were mainly Missense_Mutations and Multi_Hit (Fig. 9e, f).

### Correlation analysis of risk score with tumor immune microenvironment and immune cell infiltration

**Figure 10:**
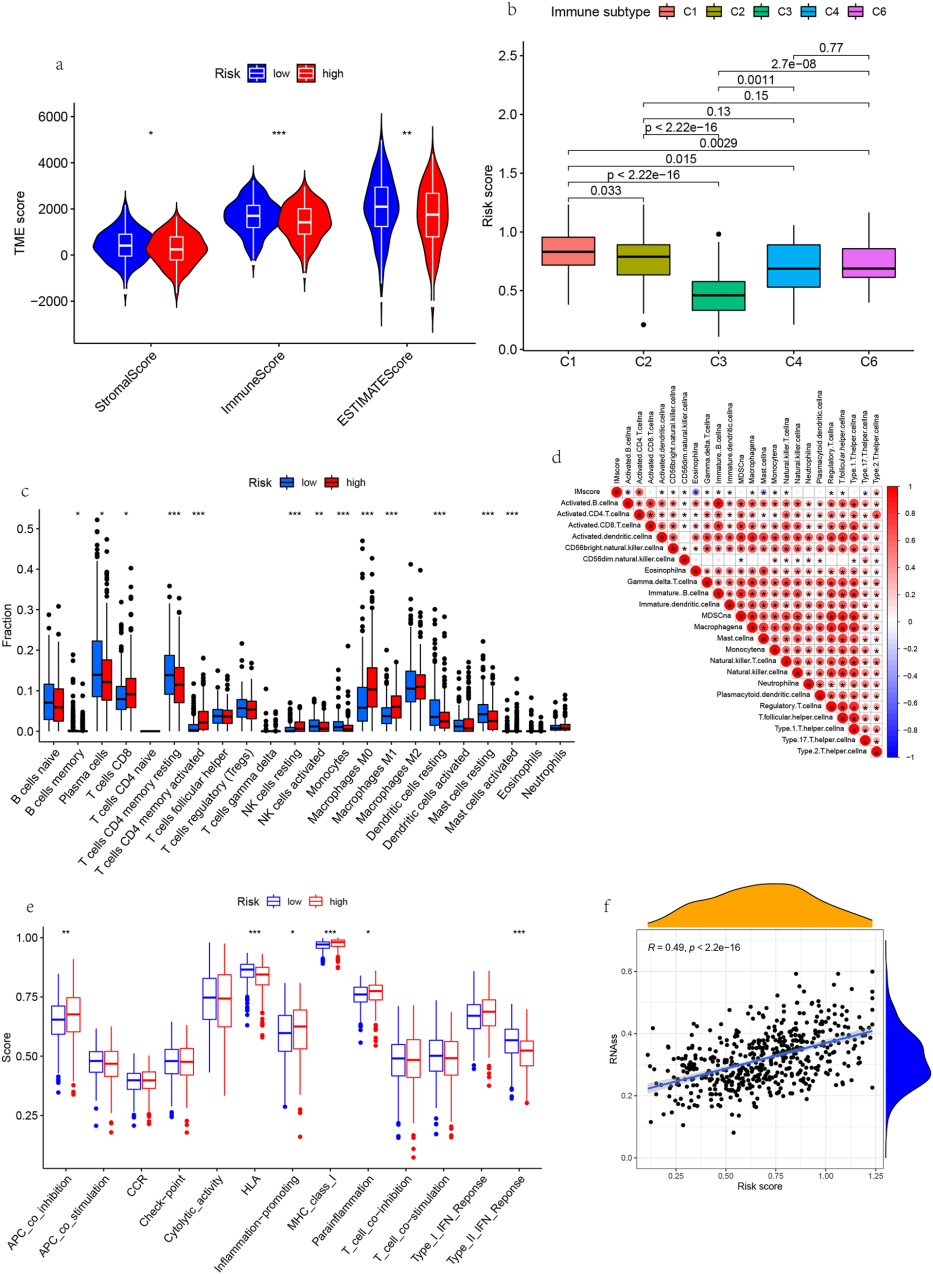
Relationship between tumor immune cell infiltration and risk score. (**a**) Relationship between tumor microenvironment and immune risk characteristics. (**b**) The relationship between risk score and immune typing. (**c**) Association between tumor infiltrating immune cells and immune risk characteristics. (**d**) Correlation analysis of immune cells. (**e**) Analysis of immune related function among different risk groups in The Cancer Genome Atlas (TCGA) cohort. The boxplot shows the scores of 13 immune related functions. The pvalue is displayed as: * P < 0.05,** P < 0.01,*** P < 0.001. (**f**) Correlation between stem cells and risk score.

In order to study the relationship between risk score and immune microenvironment, the estimate algorithm was used to quantify the matrix score, immune score, estimate score and tumor purity. The stromal score, immune score and estimate score of the low-risk group were higher than those of the high-risk group (P < 0.05,Fig. 10a). Therefore, the tumor purity of high-risk group was higher than that of low-risk group, it was associated with poor prognosis (Fig. S3a–3d) . There was significant difference between risk score and immune subtype (P < 0.05), and the risk value was the highest in C1 (Fig. 10b). Using the CIBERPORT algorithm, we calculated the proportion of 22 immune cells in each LUAD sample. Then, the difference of the proportion of immune cells between the high and low risk groups was compared. The results showed that the proportion of plasma cells, T cells CD4 memory reacting, NK cells activated, monocytes, dendritic cells reacting and mast cells resting was significantly higher in the low-risk group, and the proportion of M0 macrophases (P < 0.001), M1 macrophases (P < 0.001), T cells CD4 memory activated (P < 0.001) and NK cells resting (P < 0.001) in the high-risk group were significantly higher (Fig. 10c). They were associated with poor prognosis (Fig. S4a–4b). Immune correlation analysis showed that IMscore with activated CD4. T. cellna, Type. 2. T. helper. Cellna were positively correlated, IMscore with activated B. cellna, Eosinophilna, Mast. Cellna, Type. 17. T. helper. Cellna were negatively correlated (Fig. 10d). In further study, it was found that there was a significant difference in risk score and immune related function analysis between high-risk and low-risk groups (Fig. 10e), in which HLA (P < 0.001) and Type_II_IFN_ Reponse (P < 0.001) were activated in low-risk group, MHC_class_I (P < 0.001), APC_co_inhibition (P < 0.01), Inflammation-promoting (P < 0.05), Parainflammation (P < 0.05) were mainly activated in high-risk group. MHC_class_I and Parainflammation were associated with poor prognosis (Fig. S4c–4d). The content of stem cells was positively correlated with the risk score of patients (r = 0.49, p < 2.2e−16, Fig. 10f).

### Correlation analysis between risk score, immune checkpoint and drug sensitivity

**Figure 11:**
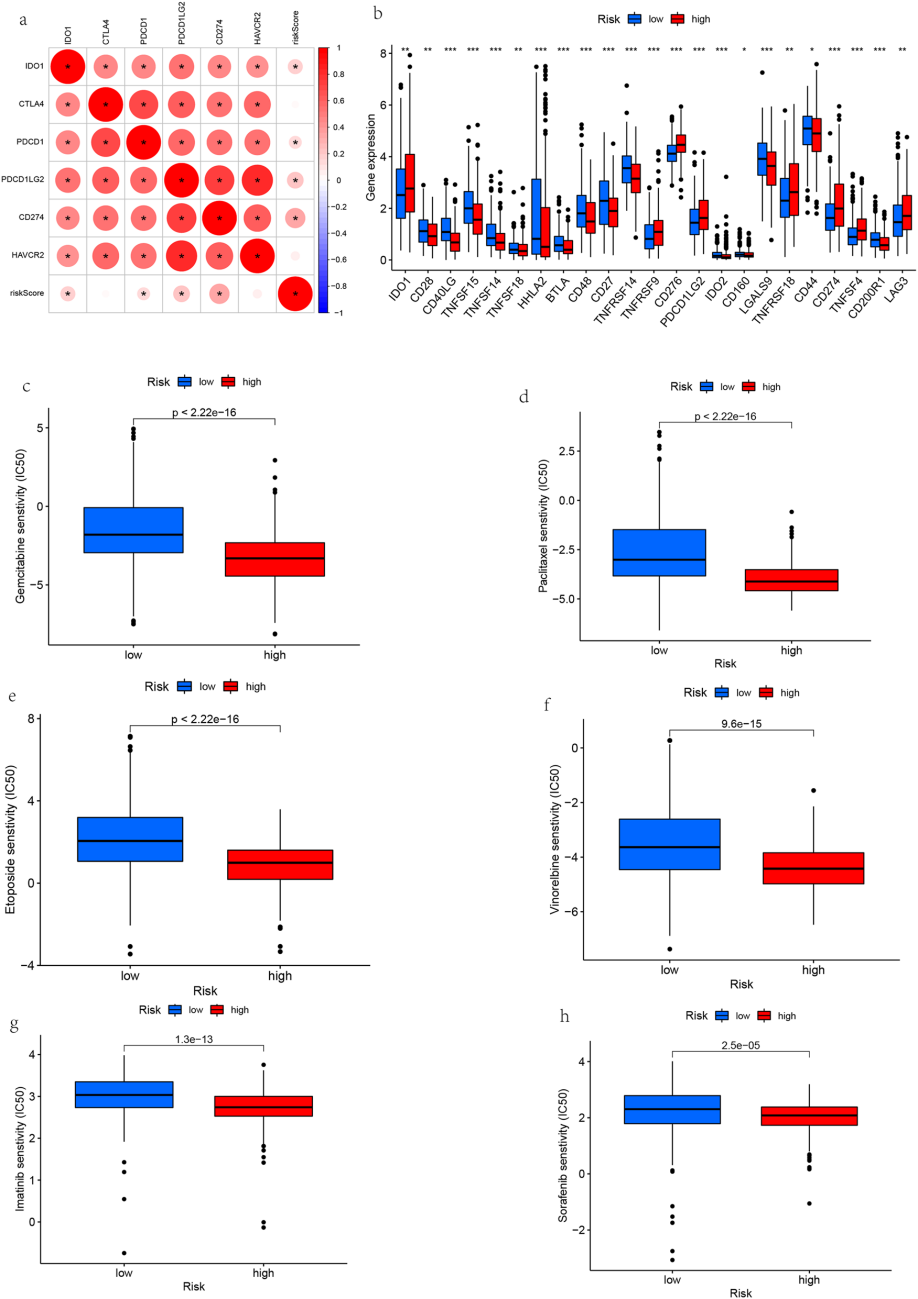
Difference analysis of immune checkpoints and screening of sensitive drugs. (**a**) Correlation between risk score and immune checkpoint. (**b**) Immune checkpoint difference analysis. (**c**–**h**) Risk score and anticancer drug sensitivity analysis.

Immune checkpoint inhibitor is a new strategy for the treatment of lung cancer in recent years. The correlation analysis of immune checkpoints showed that *CD274, PDCD1LG2, PDCD1, IDO1* were positively correlated with risk scores (Fig. 11a). The difference analysis of immune checkpoints showed that *CD40LG (P* < *0.001), TNFSF14 (P* < *0.001), TNFSF15 (P* < *0.001), CD48 (P* < *0.001), CD27 (P* < *0.001)* were highly expressed in the low-risk group, *TNFRSF9 (P* < *0.001), CD276 (P* < *0.001), PDCD1LG2 (P* < *0.001), CD274 (P* < *0.001) and TNFSF4 (P* < *0.001)* were highly expressed in the high-risk group (Fig. 11b). CD274 was highly expressed in the high-risk group, thus, the high-risk group was more suitable for anti-PD-L1 treatment. Semi-inhibitory concentration (IC50) is an important index to evaluate the efficacy or response of drugs. We studied the risk score and the sensitivity of anticancer drugs, and found that the risk score is related to many anticancer drugs, such as *gemcitabine, paclitaxel, etoposide, vinorelbine, imatinib, sorafenib,* among others, which are more suitable for high-risk patients. These results suggest that the risk score can be used as a potential predictor of chemotherapy sensitivity, providing new insights for the treatment of tumors and the prevention of drug resistance (Fig. 11c–h).

### Validate model accuracy in GEO datasets

**Figure 12:**
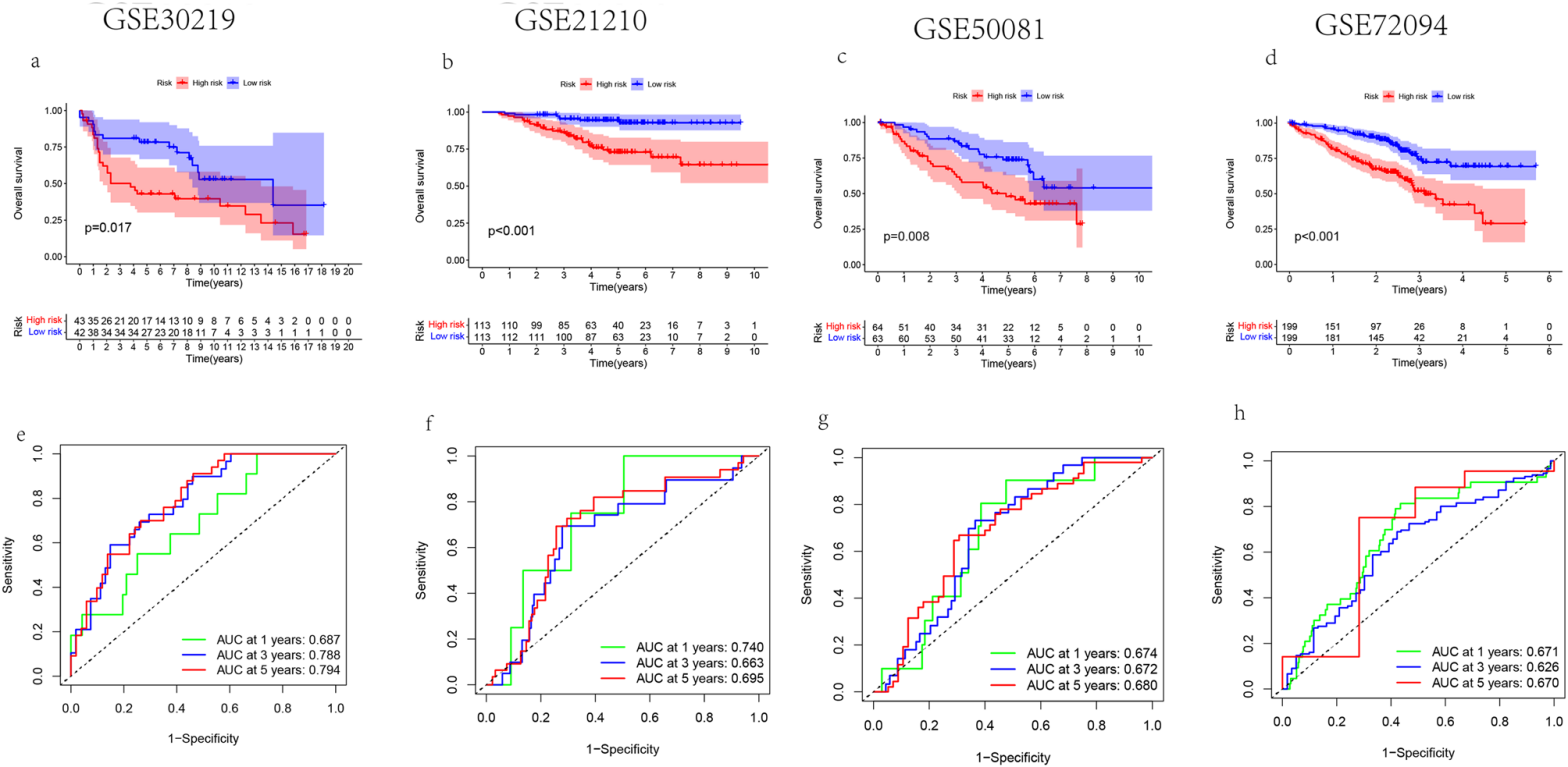
Model validation. (**a**–**d**) Kaplan Meier OS curve of Gene Expression Omnibus (GEO) datasets. (**e**–**h**) Receiver operating characteristic (ROC) curve analysis of GEO datasets.

To determine the predictive power of the six gene prognostic model in other datasets, four LUAD patient datasets (GSE30219, GSE31210, GSE50081, GSE72094) as external validation. The same formula was used to calculate the risk score of patients in the GEO cohort. According to the optimal threshold, LUAD patients were divided into high-risk group and low-risk group. The survival curve showed that patients in the high-risk group had a shorter survival time (Fig. 12a–d). ROC curve was used to evaluate the sensitivity of prognostic model (Fig. 12e–h). Therefore, through these four datasets, the correctness and feasibility of the prognosis model are verified. Our model was helpful to predict the prognosis of the LUAD patients.

## Discussion

The risk model we constructed shows that there is a significant difference in prognosis between high-risk and low-risk groups. In order to further study the potential causes of poor survival outcomes in high-risk patients, we compared the immune cell infiltration, immune checkpoint gene expression and TMB in high-risk and low-risk patients, and found that the degree of tumor immune cell infiltration, the difference in immune checkpoint gene expression, and tumor mutation load may be the potential mechanisms that affect the prognosis of patients.

Tumor associated macrophages (TAMs) are important components of the tumor microenvironment (TME)^12^ and are potential targets for tumor immunotherapy^13^. We found that M0 and M1 macrophages were heavily infiltrated in the high-risk group. Macrophages are the first line of defense against pathogens and play an important role in stress response, tissue repair, and remodeling^14^. A close relationship has been reported between the degree of macrophage infiltration and poor prognosis of patients^15^, and with accelerated angiogenesis, tumor cell invasion, infiltration, and distant metastasis^16^. Macrophages can be polarized into a tumor-promoting phenotype during lung tumor progression^17^. The progression of most tumors from benign to malignant is accompanied by a significant increase in vascular density, a process known as “angiogenesis transition”^18^. Macrophages play an important role in this complex vascular remodeling^19,20^. Macrophages can produce vascular endothelial growth factor (VEGF) in human and mouse breast tumors^19,20^. When macrophages are exposed to interleukin-4 (IL-4), they express VEGF and epidermal growth factor (EGF), thus accelerating tumor angiogenesis and breast cancer metastasis^21^, leading to poor prognoses.

The activation of PD-1 and its ligand programmed cell death ligand-1 (PD-L1 or CD274) axis mediates T-cell dysfunction and failure^22^, causing tumor cells to escape immune surveillance, thus promoting tumor cell proliferation^23^. Our study showed that PDL-1 was highly expressed in the high-risk group. A previous study demonstrated that the high expression of (PD-L1) was closely related to prognosis in patients with Non-small-cell lung cancer(NSCLC)^24^, Similar conclusions were also reported for liver cancer^25^. The high expression of PD-L1 can also enhance immune checkpoint blockade (ICB) in the treatment of NSCLC^26^, urothelial carcinoma^27^.

Studies have shown that TMB can predict the efficacy of PD-1 combined with CTLA-4 blockade in patients with NSCLC^28^. In our study, the high-risk group had higher TMB. TMB was also shown to be positively correlated with response to ICB in 27 cancers^29^, and is gradually emerging as a potential marker for the same. Patients with high TMB in NSCLC are more likely to benefit from ICB therapy^30^. In our study, *TP53* mutations were significantly more frequent in the high-risk group, and are generally associated with poor prognoses^31^, Meanwhile, patients with *TP53* mutations also reportedly respond better to ICB therapy^32^. This supports our results in that the higher the expression of PDL-1, tumor mutation load, and frequency of *TP53* mutation, the greater is the sensitivity of the high-risk group to immune checkpoint inhibitors. Moreover, these results may also partly explain the underlying mechanism of poor prognosis in high-risk groups.

Among the six genes (*PLK1, HMMR, ANLN, SLC2A1, SFTPB*, and *CYP4B1*) in the prognosis model, four genes (*PLK1, HMMR, ANLN, SLC2A1*) were risk factors and two genes (*CYP4B1* and *SFTPB*) were protective factors. *PLK1* (polo-like kinase) is a member of a new serine/threonine protein kinase family^33^, and has been shown to be highly expressed in human cancers. Its overexpression is related to poor prognoses in cancers such as neuroblastoma^34^, rectal cancer^35^, and epithelial ovarian cancer^36^. Research showed that inhibition of PLK1 can up regulate the expression of PD-L1. The combination of PD-L1 blocker and PLK1 inhibitor can produce synergistic effect in mice, significantly reduce the tumor burden and prolong the survival period of mice^37^.The proliferation of tumor cells can be inhibited by inhibiting the expression of *PLK1*, which may thus be a potential target for cancer therapy^38^. Hyaluronic acid mediated motor receptor (HMMR) is an extracellular matrix component that is closely related to cell proliferation^39^. It is associated with poor prognoses and is overexpressed in various cancers such as pancreatic cancer^40^, bladder cancer^41^, and glioblastoma^42^,among others. HMMR was associated with the reduction of the overall survival of lung cancer patients. In addition, it can pass HCG18/miR-34a-5p/HMMR axis that accelerate the progression of lung adenocarcinoma^43^. *ANLN* is an actin binding protein that is associated with poor prognosis and is highly expressed in many malignant tumors such as pancreatic cancer^44^,LUAD^45^, and nasopharyngeal carcinoma^46^,among others. ANLN played a key role in human lung cancer by participating in phosphoinositide 3-kinase/AKT pathway. Selective inhibition of ANLN may be a new strategy for the treatment of lung cancer^47^.Solute carrier family 2 member 1 (*SLC2A1*), also known as glucose transporter 1 (*GLUT1*), is a glucose transporter coding gene related to the growth and proliferation of tumor cells^48^. Its overexpression is s imilarly related to poor prognosis in cancers such as colorectal cancer^49^,breast cancer^50^,and pancreatic cancer^51^, among others. It has a particularly essential role in the occurrence and progression of tumors, and may be one of the driver genes of lung cancer^52^. Surfactant protein B (SFTPB), secreted by type II alveolar epithelial cells, is the main component of pulmonary surfactant^53^, and its precursor form can predict the risk of lung cancer^54^. *CYP4B1* is a cytochrome P450 monooxygenase. The loss of *CYP4B1* gene expression is related to bladder urothelial carcinoma^55^, and its low expression is related to the poor prognosis of LUAD patients. Therefore, it can be used as an independent prognostic marker and a potential therapeutic target for patients with LUAD^56^.

All in all, this study used WGCNA to identify the module genes related to immunotherapy, and screened out the genes related to prognosis through differential analysis and univariate Cox regression. Through consensus classification, patients were divided into three clusters. Subsequently, 125 DEGs were identified after the intersection of the three clusters. Six key genes were determined to construct a prognosis model through univariate Cox regression analysis and LASSO analysis. Patients were divided into high-risk and low-risk groups. Through analysis and comparison, patients in high-risk and low-risk groups had significant differences in prognosis, tumor immune microenvironment, tumor mutation burden, immunotherapy and immune checkpoints. Finally, the validity of the prediction model was successfully verified in the dataset of four external queues (GSE30219, GSE31210, GSE50081, GSE72094).These findings may provide new ideas for the treatment of lung cancer. However, this study still has some limitations. Our research was only based on the public database, which requires a larger sample size and further experiments to verify the predictive ability of the prognosis model. In addition, the role of key genes in the model also needs to be verified by a large number of experiments.

## Conclusion

In conclusion, Our study has constructed a prediction model based on 6 genes, which divided LUAD patients into high-risk and low-risk groups. The IMscore played an important role in predicting clinical prognosis and sensitivity to anti-tumor drug treatment, which may help us to provide new strategies for personalized treatment of LUAD patients.

## Supplementary Information

Supplementary Figures.

Supplementary Legends.

Supplementary Tables.

## Data Availability

All data were publicly available from TCGA (https://portal.gdc.cancer.gov/) and GEO (https://www.ncbi.nlm.nih.gov/geo/) datasets. These data are available from the corresponding author upon reasonable request.
