# Supplementary figures and images for: Analysis of prognostic model based on immunotherapy related genes in lung adenocarcinoma
Source: Sci Rep. 2022 Dec 21;12:22077. doi: 10.1038/s41598-022-26427-0 (PMC9772350; doi:10.1038/s41598-022-26427-0)

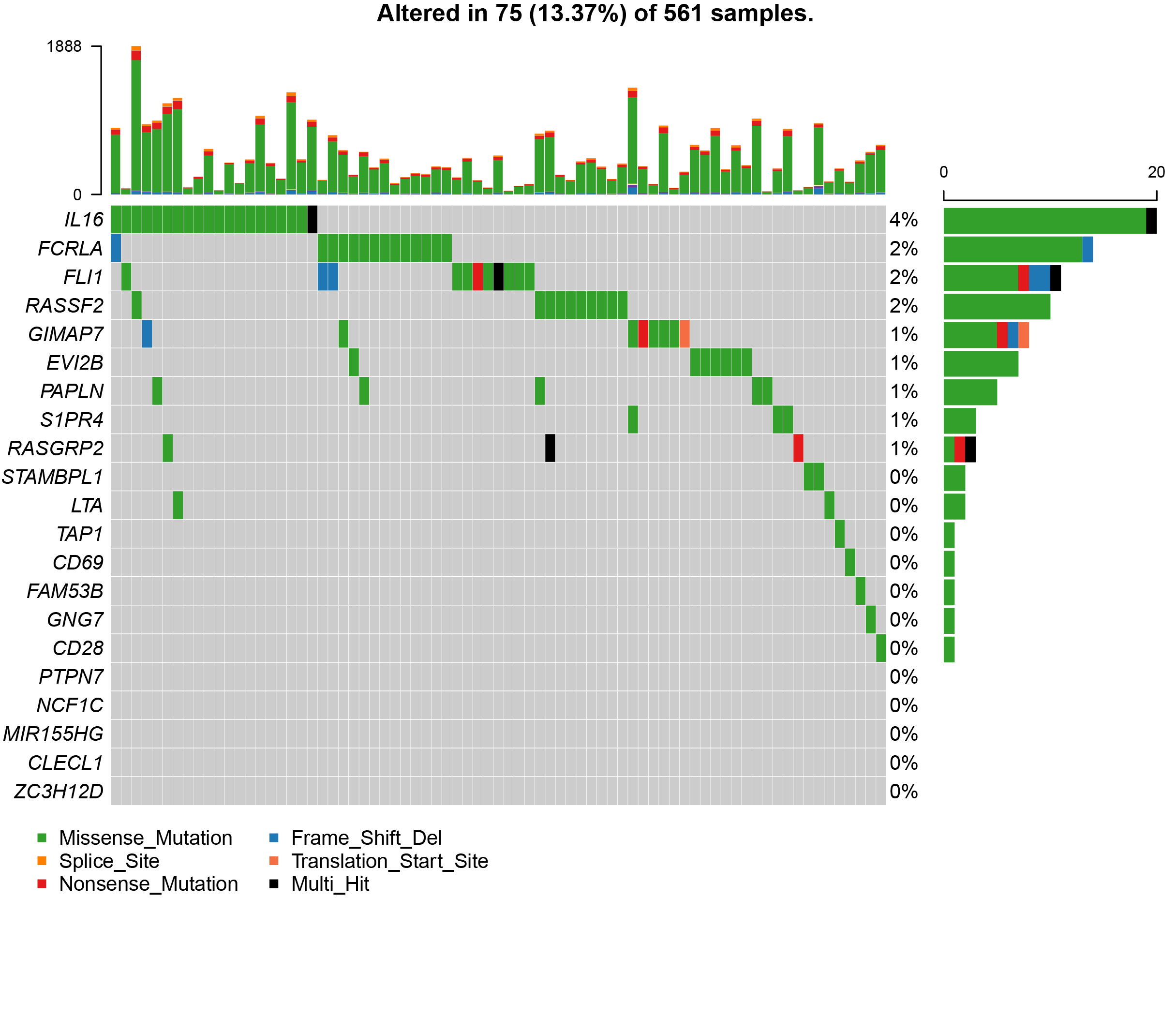

Supplement: Supplementary file 1 — Supplementary Figures. [file 41598_2022_26427_MOESM1_ESM.zip › Supplementary Figures/Supplementary Fig S1.tif]

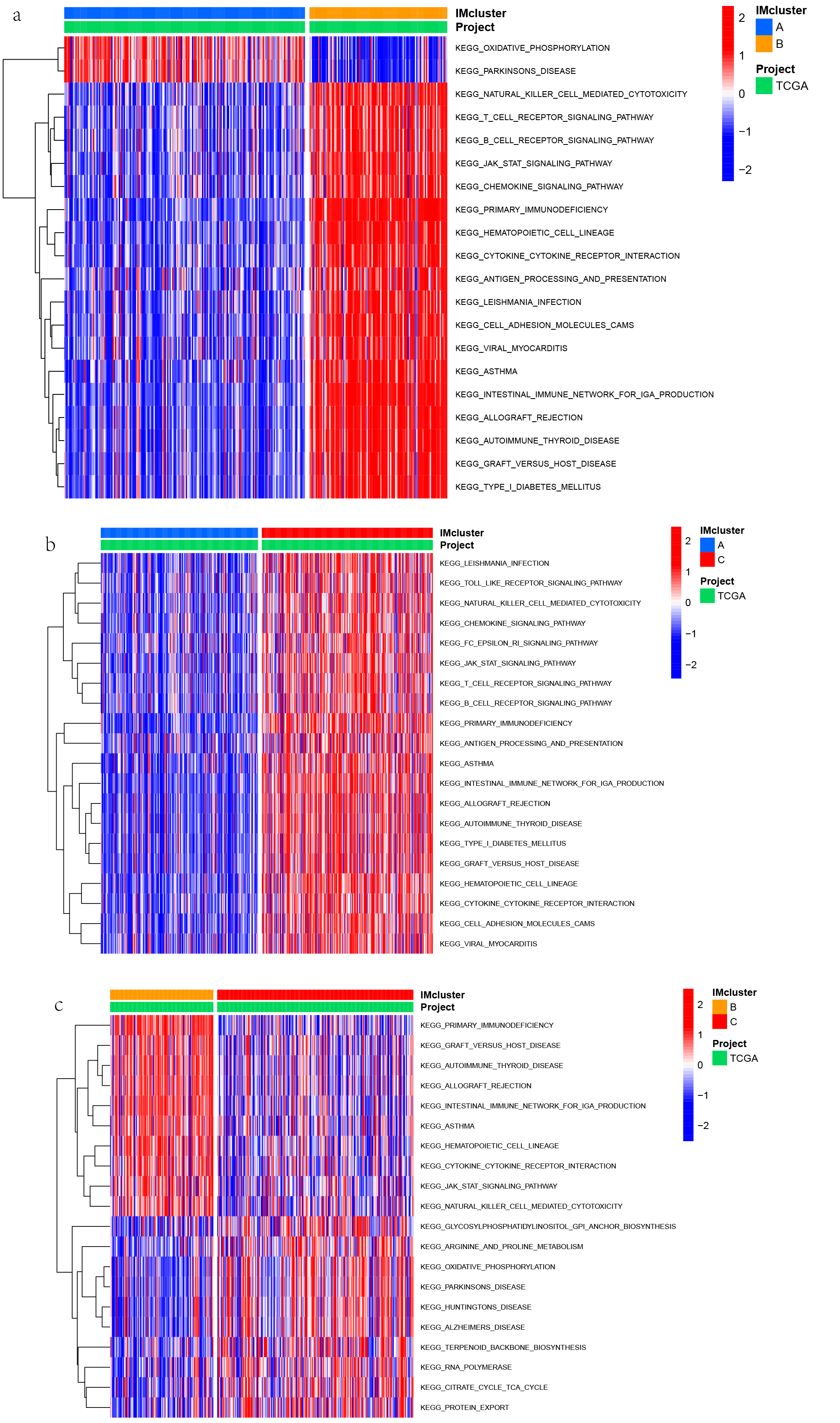

Supplement: Supplementary file 1 — Supplementary Figures. [file 41598_2022_26427_MOESM1_ESM.zip › Supplementary Figures/Supplementary Fig S2.tif]

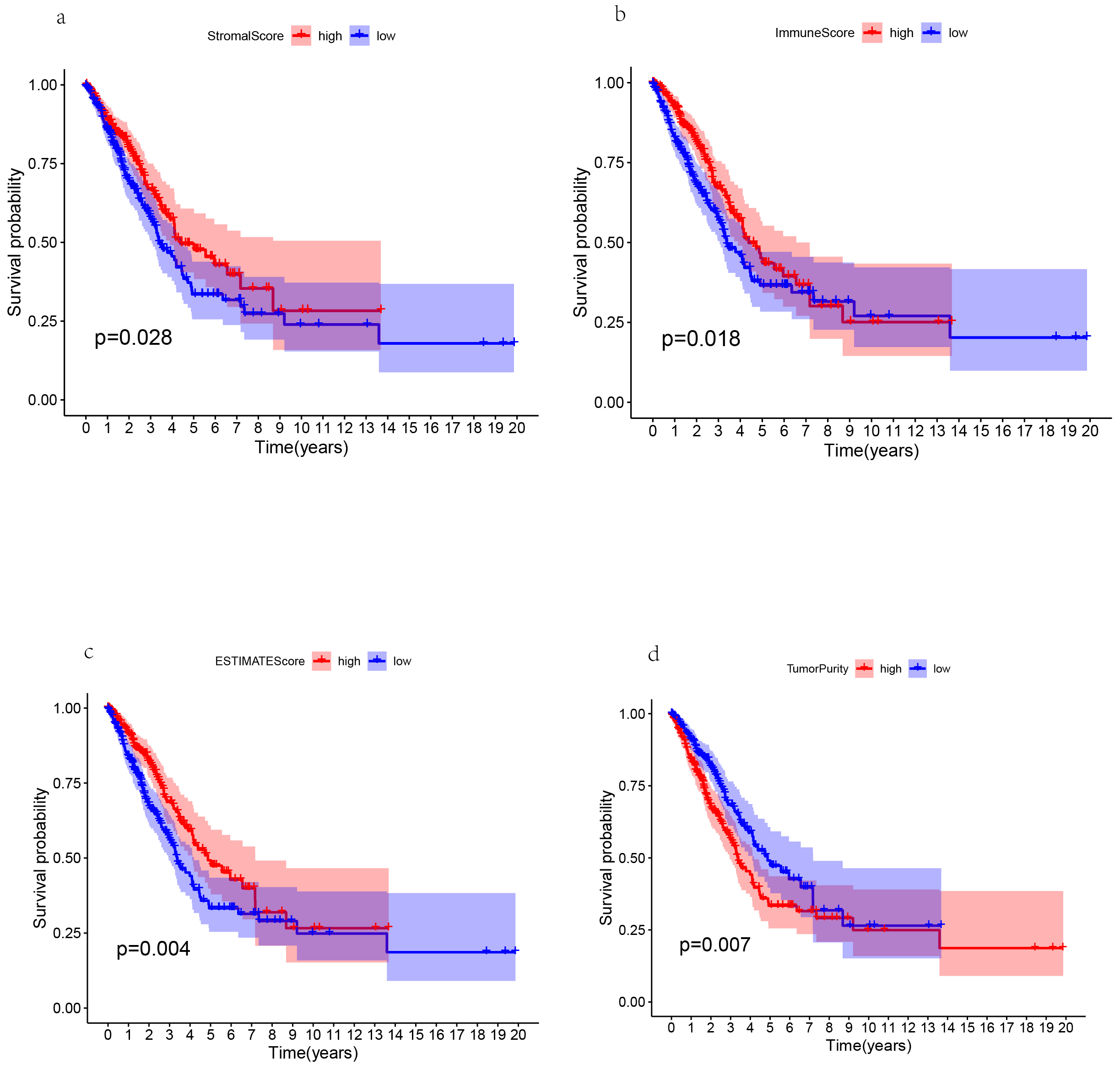

Supplement: Supplementary file 1 — Supplementary Figures. [file 41598_2022_26427_MOESM1_ESM.zip › Supplementary Figures/Supplementary Fig S3.tif]

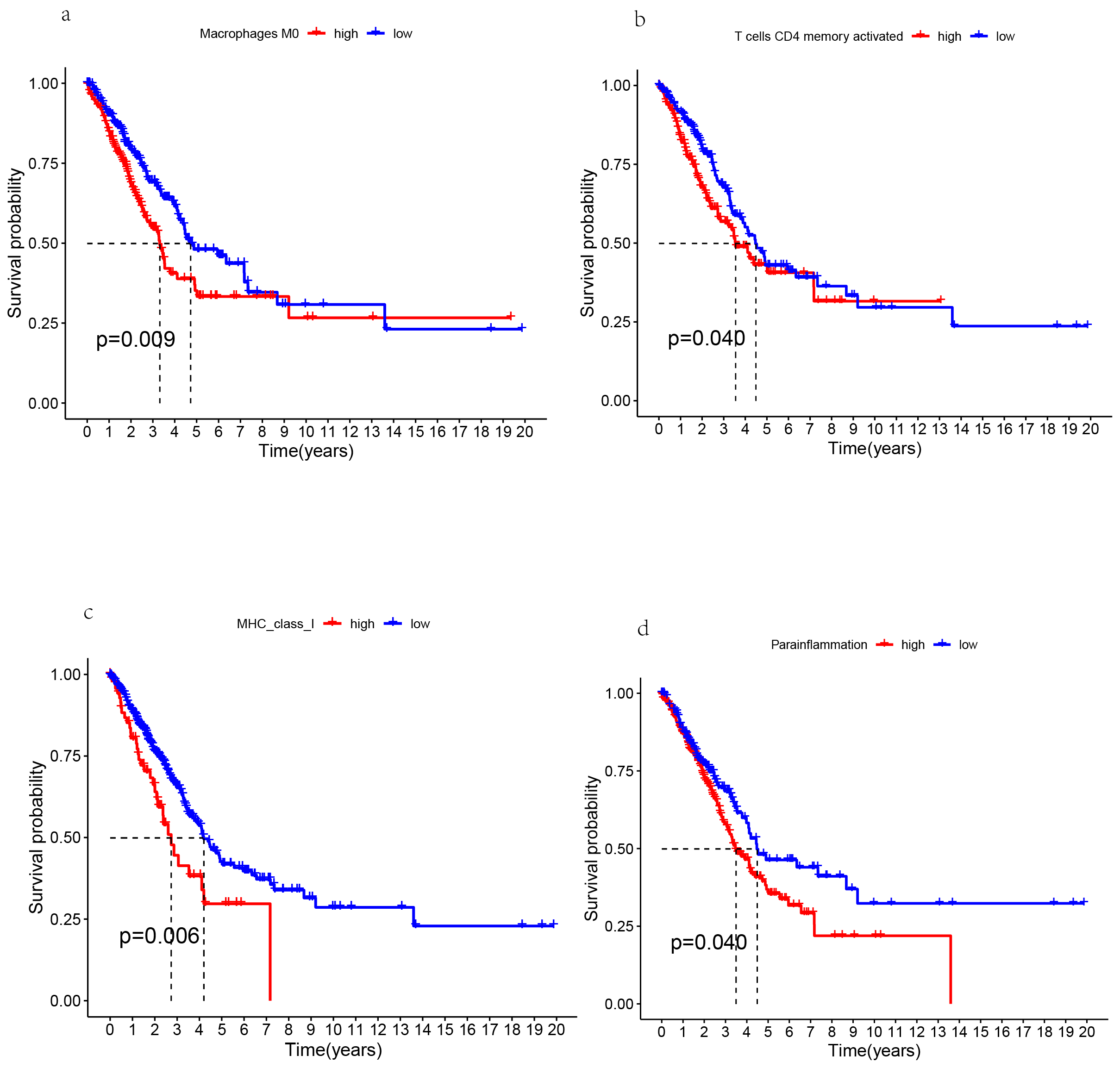

Supplement: Supplementary file 1 — Supplementary Figures. [file 41598_2022_26427_MOESM1_ESM.zip › Supplementary Figures/Supplementary Fig S4.tif]
