# Supplementary material for: Analysis of prognostic model based on immunotherapy related genes in lung adenocarcinoma
Source: Sci Rep. 2022 Dec 21;12:22077. doi: 10.1038/s41598-022-26427-0 (PMC9772350; doi:10.1038/s41598-022-26427-0)
Supplement: Supplementary file 2 — Supplementary Legends. [file 41598_2022_26427_MOESM2_ESM.docx]

Figure captions.

Fig. S1:Mutation of prognosis related genes.

Fig. S2:**Enrichment analysis among clusters**. （a）Enrichment analysis between cluster A and cluster B. （b）Enrichment analysis between cluster A and cluster C. （c）Enrichment analysis between cluster B and cluster C.

Fig. S3:OS curve of each immune score.

Fig. S4:OS curve of immune cells and immune function.
